# Beyond burden metrics: Wearable photoplethysmography-derived spatiotemporal progression of atrial fibrillation linked to clinical outcomes

**DOI:** 10.1016/j.hroo.2025.12.020

**Published:** 2026-01-08

**Authors:** Yutao Guo, Hong Wang, Hao Wang, Hui Zhang, Zhigeng Jin

**Affiliations:** 1 Pulmonary Vessel and Thrombotic Disease, Sixth Medical Center, Chinese PLA General Hospital, Beijing, China; 2 Department of Cardiology, Second Medical Center, Chinese PLA General Hospital, Beijing, China

**Keywords:** Atrial fibrillation, Wearable, Photoplethysmography, Spatiotemporal, Progression

## Abstract

**Background:**

Traditional atrial fibrillation (AF) classification lacks dynamic quantification. Current AF burden assessment is constrained by intermittent monitoring and simplistic metrics.

**Objective:**

This study aimed to establish a continuous, multidimensional AF progression model using wearable photoplethysmography (PPG) for real-world, dynamic burden quantification.

**Methods:**

In this prospective cohort, 110 patients with paroxysmal AF underwent synchronized PPG (Huawei Watch GT3) and 24-hour Holter monitoring. We developed a multiscale fusion AF algorithm and a 5-dimensional spatiotemporal progression model quantifying episode frequency, duration, clustering, circadian rhythm, and tachycardia burden.

**Results:**

The fusion algorithm achieved an accuracy of 0.97. The 5-dimensional model showed strong concordance with Holter monitoring, with near-perfect correlation for episode duration (r = 0.97) and high interchangeability (intraclass correlation coefficient >0.75). It demonstrated excellent diagnostic performance for burden trajectories (area under the curve 0.98). A composite AF burden score of ≥0.59 identified patients at high risk of AF-related symptoms or heart rate issues. Clinically, AF burden increased with worsening European Heart Rhythm Association symptoms (*P* = .04), varied by risk profile (*P* = .03), and differed between ablation- and drug-treated patients (episode duration *P* < .001).

**Conclusion:**

Continuous PPG-based spatiotemporal modeling robustly quantified dynamic AF progression, enabled precise phenotyping, and may support early intervention in high-risk patients.

## Graphical abstract

**Figure:**
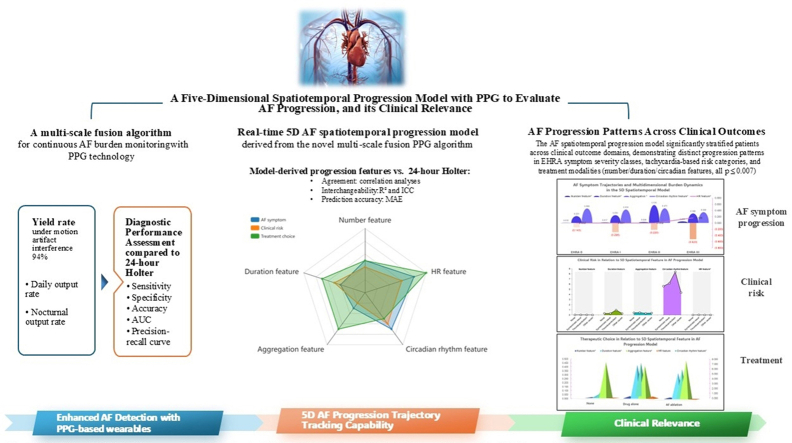

**Key Findings**

▪ Traditional classification of atrial fibrillation (AF) relies on episodic, binary assessments that fail to quantify its dynamic progression, limited by intermittent Holter monitoring, invasive implants, and simplistic burden metrics.
▪ Combining a motion-robust photoplethysmography (PPG) algorithm with a novel spatiotemporal progression model, this strategy facilitated real-time, multidimensional tracking of AF dynamics.
▪ We developed the first PPG-based spatiotemporal AF progression model, which achieved 98% area under the curve for burden trajectory prediction by quantifying 5 key dimensions of episode dynamics: frequency, duration, temporal clustering, circadian rhythm, and tachycardia burden.
▪ AF duration burden (episodes >6 minutes) increased with worsening European Heart Rhythm Association symptoms (χ^2^ = 8.33; *P* = .04), tachycardia burden differed between thromboembolic and rhythm disorder risk profiles (χ^2^ = 10.8; *P* = .03), and ablation patients exhibited distinct progression in episode duration (*P* < .001) and circadian rhythm (*P* = .002) compared with those on drug therapy.
▪ A composite AF burden score of ≥0.59 identified patients at high risk of clinically relevant AF manifestations, specifically AF-related symptoms or heart rate control issues.

## Introduction

Atrial fibrillation (AF), a prevalent arrhythmia affecting more than 60 million people globally, imposes a growing health care burden through stroke, heart failure, and mortality.^1^

AF progression remains inadequately captured by conventional clinical classifications, paroxysmal, persistent, and permanent AF, which rely heavily on symptomatic patient reports and snapshot electrocardiogram (ECG) findings during medical visits. This approach delineates the dynamic trajectory. In contrast, clinical risk scores, such as the CHA_2_DS_2_-VASc score, cannot differentiate risk variations among patients with identical scores but divergent AF patterns. For instance, a patient with paroxysmal AF and frequent short bursts may face a higher thrombotic risk than one with infrequent prolonged episodes, yet current guidelines offer no clear guidance here owing to insufficient evidence.

The concept of AF burden has emerged to refine risk assessment by correlating arrhythmia duration with clinical outcomes.^2^ Some studies observed that “AF duration” monitored by cardiac implantable electronic devices influenced stroke risk and treatment response.^3^^,^^4^ However, AF burden lacks a universal definition, usually defined as time in AF or the longest episode duration.^5^ Recent studies report divergent AF burden thresholds, from minutes to hours, for stroke risk escalation. For example, AF burden of >5–6 minutes was associated with a 2.4-fold increased stroke risk in the SOS-AF project; AF burden of >5.5 hours daily escalated stroke risk in the TRENDS study, but only prolonged episodes (>24 hours) significantly increased stroke risk in the ASSERT trial.^6^, ^7^, ^8^

Moreover, the clinical utility of the current AF burden is hampered by methodological limitations. Intermittent ECG monitoring (eg, Holter) possibly underestimated AF burden by >20%.^9^ Short AF episodes (<6 minutes) were poorly detected by cardiac implantable electronic devices, leading to underestimation.^10^ This disparity in monitoring sensitivity obscures the precise relationship between AF burden and complications such as stroke or heart failure.

Photoplethysmography (PPG) technology has achieved AF detection accuracy comparable with that of ECGs,^11^, ^12^, ^13^ offering unprecedented accessibility and continuity in arrhythmia tracking. A recent meta-analysis showed that smartwatches equipped with PPG technology can quantify AF burden as an alternative to reference ECG methods, even across heterogeneous clinical settings and patient populations.^5^ However, motion artifacts often lead to noninterpretable PPG signals, which are reported in up to 50.7% of cases in some studies.^14^ In addition, AF progression may involve complex spatiotemporal dynamics that extend beyond time-based burden metrics.

Therefore, we aimed to investigate spatiotemporal AF dynamics beyond time-based burden metrics, developed a PPG-based spatiotemporal progression model integrating novel motion-tolerant algorithms and multidimensional features, and validated this model against gold-standard monitoring and evaluated its correlation with clinical risk stratification.

## Methods

### Study design and participants

This prospective diagnostic accuracy cohort study consecutively enrolled adults (≥18 years) with documented paroxysmal AF confirmed by 12-lead ECG or 24-hour Holter monitoring. Recruitment occurred at the Sixth Medical Center, Chinese PLA General Hospital, Beijing, China, between January 1, 2024, and May 30, 2025. The study protocol received approval from the ethics committee of the institutional review board of the Chinese PLA General Hospital (HZKY-PJ-2023-23) and was registered with the Chinese Clinical Trial Registry (ChiCTR2300075516). All participants provided a written informed consent before enrolment. The research reported in this paper adhered to the Declaration of Helsinki.

We included participants aged 18 years or older with a documented diagnosis of paroxysmal AF (confirmed by the 12-lead ECG or the 24-hour Holter) who provided a written informed consent. We excluded participants with contraindications or inability to operate wearable devices, documented cognitive impairment, or the presence of implanted electronic cardiac devices (such as pacemakers or defibrillators).

### Procedures

We conducted continuous monitoring of AF progression in adults with documented paroxysmal AF using synchronized PPG-based wearable technology (Huawei Watch GT3; Huawei Technologies Co, Ltd, Shenzhen, China) and 24-hour ambulatory Holter.

Participants wore the smartwatch continuously, enabling minute-by-minute AF burden quantification. Synchronized 24-hour Holter monitoring (the reference standard) was performed at enrolment. Baseline characteristics were recorded for all enrolled patients.

AF progression trajectory tracking comprised 3 sequential stages: (1) PPG signal acquisition and preprocessing, (2) multiscale fusion algorithm development, and (3) 5-dimensional (5D) spatiotemporal progression modeling. A detailed schematic of the model workflow is provided in Supplemental Figure 1.

Clinical follow-up visits were scheduled at the Sixth Medical Center, Chinese PLA General Hospital, and additionally triggered by patient-reported symptoms (eg, palpitations, dyspnea) or arrhythmia-related events. During these visits, we assessed (1) symptom evolution using the European Heart Rhythm Association (EHRA) classification, (2) clinical risk profiles (eg, thromboembolism, rhythm disorders), and (3) treatment decisions (escalation to antiarrhythmic drug therapy or catheter ablation).

Patient-reported symptom severity (EHRA class) was recorded at the visit. Physiological data from the wearable (PPG waveforms, heart rate) were continuously streamed and processed using the multiscale fusion AF algorithm and the 5D spatiotemporal progression model to quantify AF episode frequency, duration, temporal clustering, circadian rhythm patterns, and tachycardia burden (Supplemental Figure 2).

### Multiscale fusion algorithm for continuous AF burden monitoring

To overcome limitations of existing single-episode AF detection algorithms, notably susceptibility to motion artifacts and low signal yield during activity, which impede reliable AF burden assessment, we developed a novel multiscale fusion algorithm.

This algorithm processed data in per-minute epochs and integrated 3 core components: (1) signal quality optimization (preprocessing extracted usable PPG waveforms from motion-corrupted data), (2) length-adaptive machine learning architecture (dynamically accommodated variable-duration PPG segments for robust classification), and (3) contextual fusion mechanism (leveraged the temporal continuity of AF episodes by integrating classification results from preceding and subsequent epochs to calibrate individual epoch assessments and enhance accuracy).

This integrated framework quantified AF burden as the percentage of valid monitoring time spent in AF.

### 5D AF spatiotemporal progression model

Building upon the multiscale fusion algorithm, we developed a model to quantify key dynamics of AF progression. This model captured 5 distinct dimensions:

Episode frequency: features included instantaneous AF burden ratio (AF epochs/total epochs), short-term AF episode ratio (episodes ≥6 min/total epochs), and extended AF episode ratio (episodes ≥1 h/total epochs) (Supplemental Figure 3).

Duration patterns: features included overall AF burden (total AF duration/total time), burden of sustained AF episodes (duration of episodes ≥6 min/total time), and burden of prolonged AF episodes (duration of episodes ≥1 h/total time) (Supplemental Figure 4).

Temporal clustering: feature quantified burst-like concentration patterns of AF episodes (Supplemental Figure 5).

Circadian rhythm: quantified the temporal distribution and stability of AF patterns using 5 validated metrics: (1) coefficient variation (CV) of AF episode onset (reflected measures dispersion of episode start times across 24-hour cycles), (2) the variation of the mean for AF burden (quantified rhythm robustness), (3) AF variability (reflected day-to-day fluctuation in AF burden), and (4) average real variability (calculated short-term fluctuation in hourly AF burden).

Tachycardia burden: quantified pulse rate abnormalities during AF using relative tachycardia duration (time >120 beats per minute [bpm]/total valid PPG time) and tachycardia episode frequency (epochs >120 bpm/total valid PPG epochs).

### Outcomes

The primary outcome was concordance between PPG-derived AF progression features (from the smartwatch/model), and the 24-hour Holter was rigorously assessed.

The secondary outcomes included the diagnostic performance of the PPG-AF progression model, the valid signal yield rate of the multiscale fusion algorithm for AF detection, and the clinical correlations linking continuous AF burden trajectories, derived from the spatiotemporal progression model, to adverse clinical endpoints (stroke, heart failure) and therapeutic decisions (ablation, drug).

### Statistical analysis

Continuous variables were presented as means ± standard deviations (SDs) if normally distributed or medians (interquartile ranges) if non-normal. Categorical variables were presented as frequencies (percentages).

#### AF detection capability of the multiscale fusion AF algorithm

The primary metric was a minute-by-minute AF yield rate, defined as the percentage of the total monitoring period where the algorithm generated a valid AF classification output, specifically evaluating tolerance to motion artifacts and signal degradation.

#### Diagnostic performance assessment against the gold standard

Performance for detecting AF per 1-minute epoch (aligned with Holter analysis) was assessed using sensitivity, specificity, accuracy, the receiver operating characteristic area under the curve, and precision-recall curves. Holter served as the gold standard.

#### AF progression tracking of AF spatiotemporal progression model vs Holter

Agreement between model-derived progression metrics and Holter-derived metrics was evaluated for each dimension using the following:

Correlation: Pearson (normal data), Spearman, or Kendall’s tau (non-normal/ordinal data), reported as correlation coefficient (R-value) and mean absolute error (MAE). $\text{MAE}=\frac{\sum_{i=1}^{n}\left|p_{i}-g_{i}\right|}{n}$, $p_{i}:PPG\;g_{i}:ECG$.

Interchangeability: coefficient of determination (R^2^) and intraclass correlation coefficient (ICC) (2-way random-effects model for absolute agreement) were applied. The R^2^ quantified the proportion of variance in Holter ECG results explained by PPG measurements, reflecting their linear correlation (values closer to 1 indicated strong alignment in trends). The ICC assessed absolute agreement by accounting for both systematic biases and random errors between methods, with values >0.75 indicating excellent consistency, 0.50–0.75 moderate, and <0.50 poor. High R^2^ (>0.8) and high ICC (>0.75) supported interchangeability.

#### A composite AF burden value synthesized from 5 progression dimensions

The final AF burden model value was computed as a weighted sum of the normalized 5D features, where the weights were derived from the integration of the correlation analysis, regression modeling, and consistency analysis. We used Youden’s index maximization method to determine the optimal cutoff value of AF burden, in relation to clinical events.

#### AF progression patterns across clinical outcomes

The Kruskal-Wallis H test assessed whether distributions of the 5 AF progression dimensions differed significantly across categories of symptom severity (EHRA class), clinical risk profiles, or treatment choices.

Statistical significance was defined as a 2-sided *P* < .05. 95% confidence intervals (CIs) were calculated using the Wilson score method without continuity correction. Analyses were performed using IBM SPSS Statistics version 25.0 (IBM Corp, Armonk, NY).

## Results

The study included 110 patients (65.5% male; mean age ± SD 62 ± 14 years) (Table 1). Over a mean follow-up of 303 ± 125 days, 45 clinical visits occurred: 33 for AF symptoms or heart rate issues, 1 for thromboembolism, and 11 for other indications. Overall, 32 patients received drug therapy alone, and 19 underwent AF ablation (Supplemental Table 1). Compared with patients who received drug therapy or AF ablation, those without treatment had significantly fewer clinical visits for AF symptoms, thromboembolism, etc (all *P* < .001) (Supplemental Table 2).

**Table 1:** AF progression tracking capability of 5D spatiotemporal progression model compared with the 24-hour Holter (n = 110)

Number feature	PPG, mean (SD)	ECG, mean (SD)	PPG, median (interquartile range)	ECG, median (interquartile range)	MAE	Pearson correlation coefficient	Pearson test, *P* value	Spearman rank correlation coefficient	Spearman test, *P* value	Kendall’s tau correlation coefficient	Kendall’s tau test, *P* value
AF episodes/total measures	0.02 (0.03)	0.00 (0.00)	0.01 (0.00–0.03)	0.00 (0.00–0.00)	0.02	0.08	.43	0.60	<.001	0.45	1.22
AF episodes over 6 min/total measures	0.00 (0.00)	0.00 (0.00)	0.00 (0.00–0.01)	0.00 (0.00–0.00)	0.00	0.28	.003	0.81	6.06	0.65	3.34
AF episodes over 1 h/total measures	0.00 (0.00)	0.00 (0.00)	0.00 (0.00–0.00)	0.00 (0.00–0.00)	0.00	0.66	3.89	0.85	1.39	0.72	1.76
Duration feature											
AF duration/total monitoring time	0.32 (0.42)	0.33 (0.44)	0.02 (0.00–0.90)	0.00 (0.00–1.01)	0.03	0.97	<.001	0.86	<.001	0.75	<.001
AF lasting over 6 min/total monitoring time	0.29 (0.40)	0.33 (0.44)	0.00 (0.00–0.80)	0.00 (0.00–1.00)	0.05	0.96	<.001	0.94	<.001	0.87	<.001
AF lasting over 1 h/total monitoring time	0.22 (0.33)	0.33 (0.45)	0.00 (0.00–0.45)	0.00 (0.00–1.00)	0.11	0.90	<.001	0.90	<.001	0.84	<.001
Aggregation feature											
AF density	0.45 (0.27)	0.32 (0.38)	0.41 (0.22–0.65)	0.00 (0.00–0.64)	0.18	0.80	<.001	0.76	<.001	0.634	<.001
Circadian rhythm feature											
CV of AF episodes	7.23 (8.77)	5.96 (9.53)	2.83 (0.40–12.7)	0.65 (0.00–8.73)	2.81	0.87	<.001	0.82	<.001	0.67	<.001
VOM of AF episodes	−0.21 (0.26)	−0.048 (0.13)	−0.19 (−0.30 to −0.08)	−0.03 (−0.08 to 0.01)	0.20	0.40	.009	0.30	.05	0.22	.04
AF variability of AF episodes	4.17 (4.98)	2.395 (3.92)	1.91 (0.14–7.07)	0.17 (0.00–3.63)	2.13	0.78	<.001	0.80	<.001	0.64	<.001
Day-AF variability	4.95 (5.79)	2.86 (4.59)	1.90 (0.15–9.10)	0.033 (0.00–4.60)	2.73	0.74	<.001	0.80	<.001	0.61	<.001
Night-AF variability	2.98 (6.18)	1.922 (5.17)	0.00 (0.00–1.71)	0.00 (0.00–0.43)	1.12	0.87	<.001	0.72	<.001	0.65	<.001
Average real variability	5.70 (6.63)	4.18 (6.51)	2.52 (0.24–10.71)	0.44 (0.00–6.40)	2.39	0.84	<.001	0.82	<.001	0.66	<.001
Tachycardia feature											
Duration of pulse rate of >120 bpm/total monitoring time	0.01 (0.08)	0.02 (0.07)	0.00 (0.00–0.00)	0.00 (0.00–0.01)	0.03	0.12	.22	0.30	.001	0.24	.001
Number of pulse rate of >120 bpm/total measures	0.01 (0.08)	0.02 (0.07)	0.00 (0.00–0.00)	0.00 (0.00–0.01)	0.03	0.12	.22	0.30	.001	0.24	.001

$\text{MAE}=\frac{\sum_{i=1}^{n}\left|p_{i}-g_{i}\right|}{n}$, $p_{i}:PPG\;g_{i}:ECG$. Day-AF variability, 6 AM to 10 PM; night-AF variability, 10 PM to 6 PM; average real variability calculated short-term fluctuation in hourly AF burden.

AF = atrial fibrillation; bpm = beats per minute; CV = coefficient variation; ECG = electrocardiogram; MAE = mean absolute error; PPG = photoplethysmography; SD = standard deviation; VOM = variation of the mean (quantified rhythm robustness); 5D = 5-dimensional.

### Enhanced AF detection capability

The motion artifacts (mean ± SD) was 30.6% ± 10.6%. The multiscale fusion AF algorithm achieved a minute-by-minute AF yield rate of 0.93 (95% CI 0.91–0.95), with daily and nocturnal rates of 0.90 (0.87–0.93) and 0.98 (0.97–0.99), respectively. Comparison of ECG and PPG waveforms is shown in Supplemental Figure 6.

Compared with the 24-hour Holter, the sensitivity, specificity, and overall accuracy of the multiscale fusion AF algorithm were 0.90 (95% CI 0.86–0.94), 0.98 (0.97–0.99), and 0.97 (0.96–0.99), respectively.

### AF progression model

The 5D AF spatiotemporal progression model showed strong concordance with the 24-hour Holter across key progression features (Table 1). Model-derived AF episode durations correlated highly with Holter measurements (Pearson r = 0.97; *P* < .001). Circadian rhythm features also correlated significantly (CV, night-AF variability, all r = 0.87; average real variability r = 0.84; all *P* < .001). AF episode clustering quantification correlated positively with Holter (r = 0.80; *P* < .001) (Table 1).

Temporal features exhibited high precision (MAE: number feature 0.02; duration feature 0.03), whereas aggregation feature accuracy was moderate (MAE 0.18) (Table 1).

Duration and circadian features (CV, night-AF variability) were interchangeable with Holter (R^2^ > 0.75; ICC > 0.75), confirming wearable PPG’s utility for long-term monitoring (Table 2).

**Table 2:** The interchangeability of 5D AF spatiotemporal progression model and the 24-hour Holter

Number feature	R^2^ (95% CI)	ICC (95% CI)
AF episodes/total measures	0.01 (0.00–0.07)	0.01 (−0.18 to 0.19)
AF episodes over 6 min/total measures	0.08 (0.03–0.25)∗	0.05 (−0.14 to 0.23)
AF episodes over 1 h/total measures	0.44 (0.28–0.70)	0.32 (0.14–0.48)
Duration feature		
AF duration/total monitoring time	0.95 (0.89–0.99)∗	0.97 (0.96–0.98)∗
AF lasting over 6 min/total monitoring time	0.92 (0.85–0.98)∗	0.95 (0.93–0.97)∗
AF lasting over 1 h/total monitoring time	0.80 (0.69–0.91)∗	0.83 (0.76–0.88)∗
Aggregation feature		
AF density	0.64 (0.44–0.80)	0.74 (0.59–0.84)
Circadian rhythm feature		
CV of AF episodes	0.76 (0.58–0.87)∗	0.86 (0.80–0.90)∗
VOM of AF episodes	0.16 (0.00–0.58)	0.24 (−0.08 to 0.51)
AF variability of AF episodes	0.62 (0.48–0.75)	0.71 (0.60–0.79)∗
Day-AF variability	0.54 (0.41–0.68)	0.67 (0.55–0.76)
Night-AF variability	0.76 (0.48–0.93)∗	0.85 (0.78–0.89)∗
ARV	0.71 (0.56–0.83)∗	0.82 (0.75–0.87)∗
Tachycardia feature		
Duration of pulse rate of >120 bpm/total monitoring time	0.01 (0.00–0.68)	0.11 (−0.07 to 0.30)
Number of pulse rate of >120 bpm/total measures	0.01 (0.00–0.68)	0.11 (−0.07 to 0.30)

R^2^ quantifies the proportion of variance in Holter ECG results explained by PPG, reflecting linear correlation (closer to 1 = stronger trend alignment). ICC evaluates absolute agreement, accounting for systematic biases and random errors, with thresholds >0.75 (excellent), 0.50–0.75 (moderate), and <0.50 (poor consistency). Day-AF variability, 6 AM to 10 PM; night-AF variability, 10 PM to 6 AM; ARV, the VOM quantified the diurnal rhythm of AF by assessing the relative difference in AF episode frequency between daytime and nighttime, which could aid in evaluating circadian rhythm patterns of AF for clinical management. ARV quantified the natural fluctuation in AF episodes over a 24-hour period, which could provide a holistic measure of arrhythmia instability.

AF = atrial fibrillation; ARV = average real variability; bpm = beats per minute; CI = confidence interval; CV = coefficient variation; ECG = electrocardiogram; ICC = interclass correlation; PPG = photoplethysmography; VOM = variation of the mean; 5D = 5-dimensional.

∗ *p* < 0.05.

**Figure 1:**
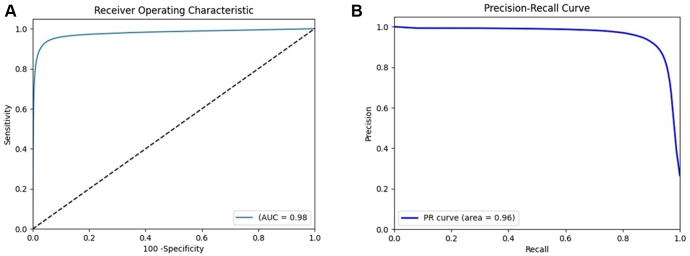
Performance of the PPG-AF progression model. **A:** The ROC-AUC performance. **B:** The precision-recall curve. ROC-AUC 0.98; 95% CI 0.97–0.99; PR curve 0.96; 95% CI 0.95–0.98; all *P* < .001. AF = atrial fibrillation; CI = confidence interval; PPG = photoplethysmography; ROC-AUC = receiver operator characteristic area under the curve.

**Figure 2:**
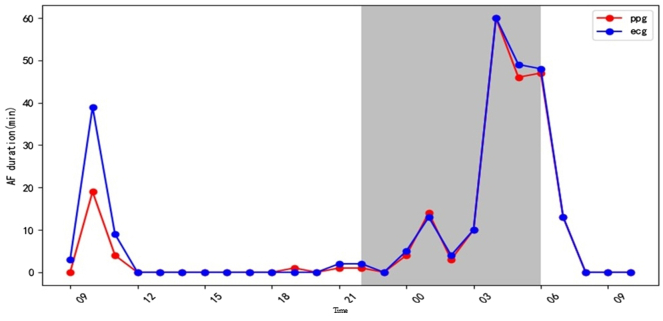
The temporal agreement in AF burden detection between the PPG-AF progression model and 24-hour ECG monitoring. *Gray zone*: night. *Blue line*: AF burden driven by PPG progression model. *Red line*: AF burden driven by ECG. AF = atrial fibrillation; ECG = electrocardiogram; PPG = photoplethysmography.

The PPG-AF progression model yielded a receiver operating characteristic area under the curve value of 0.98 (95% CI 0.97–0.99) for AF burden trajectories (Figure 1A). The precision-recall curve is shown in Figure 1B. The temporal agreement in AF burden detection between the PPG-AF progression model and 24-hour ECG monitoring is shown in Figure 2.

### AF progression patterns across clinical outcomes

**Figure 3:**
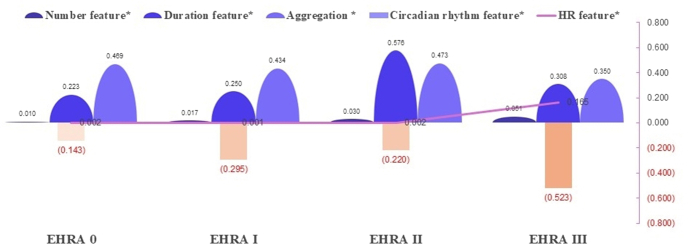
AF symptom trajectories and multidimensional burden dynamics in the 5D spatiotemporal model. Number feature, AF episodes/total measures; duration feature, AF duration/total monitoring time; aggregation feature, AF density; circadian rhythm feature, VOM of AF episodes; HR feature, duration of PPG-detected pulse rate of >120 bpm/total PPG monitoring time. ∗*P* < .05. AF = atrial fibrillation; bpm = beats per minute; EHRA = European Heart Rhythm Association; HR = heart rate; PPG = photoplethysmography; VOM = variation of the mean; 5D = 5-dimensional.

Significant differences in progression features occurred across EHRA symptom classes (I–IV; all *P* < .05) (Figure 3). The number feature increased incrementally with higher EHRA classes (*P* < .001).

Tachycardia feature proportions (>120 bpm) varied significantly across AF-related clinical risks (rhythm/rate disorders, thromboembolism, other; χ^2^ = 10.8; *P* = .03) (Supplemental Figure 7).

**Figure 4:**
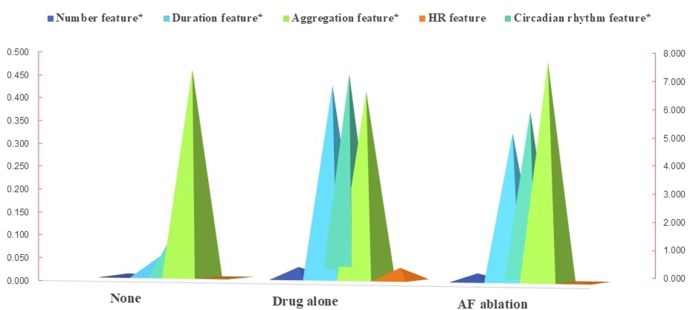
Therapeutic choice in relation to 5D spatiotemporal feature in AF progression model. Number feature, AF episodes/total measures; duration feature, AF duration/total monitoring time; aggregation feature, AF density; circadian rhythm feature, VOM of AF episodes; HR feature, duration of PPG-detected pulse rate of >120 bpm/total PPG monitoring time. ∗*P* < .05. bpm = beats per minute; AF = atrial fibrillation; EHRA = European Heart Rhythm Association; HR = heart rate; PPG = photoplethysmography; VOM = variation of the mean; 5D = 5-dimensional.

Distinct progression patterns emerged across treatment choices (number feature: no treatment vs drug therapy vs AF ablation, 0.01, 0.03, and 0.02, *P* = .007; duration feature, 0.05, 0.43, and 0.32, *P* < .001; circadian rhythm feature, 1.76, 7.24, and 5.89, *P* = .002) (Figure 4).

For AF-related symptoms or heart rate issues, the optimal diagnostic cutoff for the AF burden score was 0.59 (determined by maximizing Youden’s index), with a sensitivity of 0.80 and a specificity of 0.79 (*P* = .04).

## Discussion

We developed an integrated strategy that combines motion-robust PPG algorithms with a novel spatiotemporal progression model, enabling real-time, multidimensional tracking of AF dynamics. First, our motion-robust PPG algorithm achieved continuous, high-fidelity AF detection comparable with clinical gold standards. Second, the spatiotemporal model captured multidimensional AF progression beyond traditional classifications, showing strong concordance with reference monitoring across key physiological features. Most significantly, it identified distinct clinical progression patterns linked to symptom severity, thromboembolic risk profiles, and differential treatment choices.

To the best of our knowledge, this study represents the first comprehensive integration of continuous PPG-based monitoring with multidimensional AF progression modeling, overcoming critical limitations in current AF assessment paradigms. Our findings align with and extend previous research in several key aspects. First, we demonstrated that our motion-optimized, multiscale fusion algorithm achieved minute-by-minute AF detection (yield rate 0.93) with accuracy comparable with 24-hour Holter monitoring (sensitivity 0.90; specificity 0.98). Previous studies confirmed that even short-duration episodes contributed to cumulative risk, whereas there was also a dose-response relationship between increasing AF burden and all-cause or cardiovascular hospitalization and ischemic stroke.^6^^,^^15^ Our technological advance enabled reliable capture of clinically significant short-duration AF episodes (≥1 minute) that are frequently missed by intermittent monitoring yet contribute meaningfully to cumulative AF burden.

Second, our 5D spatiotemporal progression model addressed fundamental gaps in current AF classification by moving beyond simplistic duration-based metrics. The model demonstrated exceptional concordance with gold-standard monitoring across all progression features (r = 0.80–0.97; MAE 0.02–0.03) and revealed distinct progression patterns across EHRA symptom classes (*P* < .001) and treatment modalities, providing unprecedented granularity in tracking AF dynamics.

Higher AF burden correlated with elevated stroke risk across multiple studies, although substantial heterogeneity exists in the reported thresholds (eg, 5 minutes to >24 hours) and hazard ratios. For instance, although the KP-RHYTHM study reported a 3-fold increase in ischemic stroke risk with >11.4% AF burden,^16^ the ARTESiA and NOAH-AFNET 6 trials revealed lower event rates (∼1%/y) despite similar monitoring durations.^7^^,^^8^ This heterogeneity suggested that time-based burden alone may not fully capture the multidimensional nature of AF progression with comorbidities, underscoring the need for risk models reflecting the nature of AF progression to refine prognostic accuracy and therapeutic decision making.

The tachycardia burden of the 5D spatiotemporal progression model at >120 bpm was adopted, balancing the need to identify high-risk hemodynamic profiles with practical monitoring parameters. The lenient rate-control strategy (resting heart rate <110 bpm) is considered a safe and effective long-term approach endorsed by the guidelines,^17^ whereas sustained ventricular rates of >120–130 bpm were strongly associated with the development of tachycardia-induced cardiomyopathy. Therefore, tachycardia burden of >120 bpm was designed to flag episodes that transcend the boundaries of “controlled” AF and enter a high-risk zone for myocardial toxicity, necessitating urgent clinical reassessment. Indeed, the significant variation in the “tachycardia” feature (>120 bpm proportion) across different AF-related risk categories (rhythm/rate disorders, thromboembolic events, other etiologies) was observed. It indicated that specific facets of the spatiotemporal progression model, particularly the presence of rapid ventricular rates, were associated with distinct clinical risk profiles. This suggested the model’s potential role in risk stratification, potentially identifying patients with specific progression patterns (eg, high tachycardia burden) who might be at a greater risk of adverse events such as heart failure exacerbation or require more aggressive rate control. The model identified tachycardia-dominant profiles associated with thromboembolic risk, offering a potential complement to CHA_2_DS_2_-VASc scores in refining stroke prediction.

Third, we established that specific spatiotemporal patterns (eg, high episode density with circadian disruption) were associated with differential clinical outcomes, which was a finding that challenges the conventional focus on isolated burden thresholds. This approach proved particularly valuable in detecting subclinical progression, as evidenced by significant AF dynamics in asymptomatic patients (EHRA 0) who would typically be missed by symptom-driven monitoring.

The composite AF burden cutoff of 0.59 (determined by Youden’s index maximization) identified patients at higher risk who were more prone to developing symptoms or heart rate problems that may lead to clinical encounters. They may be more likely to benefit from early rhythm management. Our technology possibly facilitated early detection of pathologic progression patterns, potentially identifying at-risk individuals before symptom escalation. Future studies should prioritize evaluating this model as a potential tool for selecting candidates for intensive therapy.

There were several limitations in this study. First, although the number feature demonstrated an MAE, its weak correlation with the gold-standard Holter may reflect the low frequency of AF episodes in the study cohort. Although this aligned with real-world paroxysmal AF patterns, the clinical implications of infrequent but cumulative AF burden, particularly for stroke risk stratification or rhythm management, remain uncertain and warrant validation in larger cohorts with diverse AF burdens.

Second, the present study operationalized “significant” tachycardia burden using a threshold of >120 bpm. The hemodynamic impact, symptom burden, and long-term remodeling associated with heart rates between 110 bpm and 120 bpm, as well as persistent rates of >100 bpm, remain important and unresolved questions. Therefore, our findings were not generalizable to the prognostic significance or management implications of AF burden in these lower heart rate zones, which deserved future investigation.

Third, given the low event rate (n = 1), a predictive threshold of AF burden for thromboembolism could not be determined, requiring further investigation.

Fourth, the study focused on paroxysmal AF patients without implanted devices, excluding those with persistent AF or comorbidities such as pacemaker dependence. This limited extrapolation to broader AF populations, including high-risk subgroups with advanced structural heart disease.

In addition, the predominance of male participants (65.5%) and single-center design may introduce selection bias, necessitating multicenter validation to confirm the model’s robustness across demographics and clinical settings.

## Conclusion

The multiscale fusion PPG algorithm enabled continuous, high-fidelity AF monitoring with 93% detection yield and gold-standard accuracy, overcoming motion artifact limitations. The derived 5D spatiotemporal progression model captured multidimensional AF dynamics, validated against Holter monitoring, and revealed distinct clinical patterns linked to symptom severity, tachycardia burden, and treatment response. This framework bridged continuous physiological tracking with actionable phenotyping, advancing personalized AF management through accessible wearable technology. Future studies should validate its impact on long-term outcomes and therapeutic decision making.
