# Supplementary material for: Beyond burden metrics: Wearable photoplethysmography-derived spatiotemporal progression of atrial fibrillation linked to clinical outcomes
Source: Heart Rhythm O2. 2026 Jan 8;7(3):535–44. doi: 10.1016/j.hroo.2025.12.020 (PMC13031011; doi:10.1016/j.hroo.2025.12.020)
Supplement: Supplementary Material [file mmc1.docx]

**Supplementary appendix**

**Beyond Burden Metrics: wearable photoplethysmography-derived spatiotemporal progression of atrial fibrillation linked to clinical outcomes**

Yutao Guo^1^*, Hong Wang ^1^, Hao Wang^2^, Hui Zhang^1^, Zhigeng Jin^1^

1. Pulmonary Vessel and Thrombotic Disease, Sixth Medical Center, Chinese PLA General Hospital, Beijing, China, 100048
2. Department of Cardiology, Second Medical Center, Chinese PLA General Hospital, Beijing, China, 100048

**Table content**

[Figure S1. Schematic description of model flow. 2](#_Toc217205840)

[Figure S2. Multi-dimensional quantitative assessment approach of atrial fibrillation progression with photoplethysmograph-based smart watch. 3](#_Toc217205841)

[Figure S3. AF progression feature statistics schematic diagram: Episode Frequency. 4](#_Toc217205842)

[Figure S4. AF progression feature statistics schematic diagram: Duration Patterns. 5](#_Toc217205843)

[Figure S5. AF progression feature statistics schematic diagram: Temporal Clustering. 6](#_Toc217205844)

[Table S1. Patient’s characteristics at baseline. 7](#_Toc217205845)

[Table S2. Clinical visits in relation to AF treat pattern. 8](#_Toc217205846)

[Figure S6. Comparison of ECG and PPG waveforms. 9](#_Toc217205847)

[Figure S7. Clinical risk in relation to 5D spatiotemporal feature in AF progression model 10](#_Toc217205848)


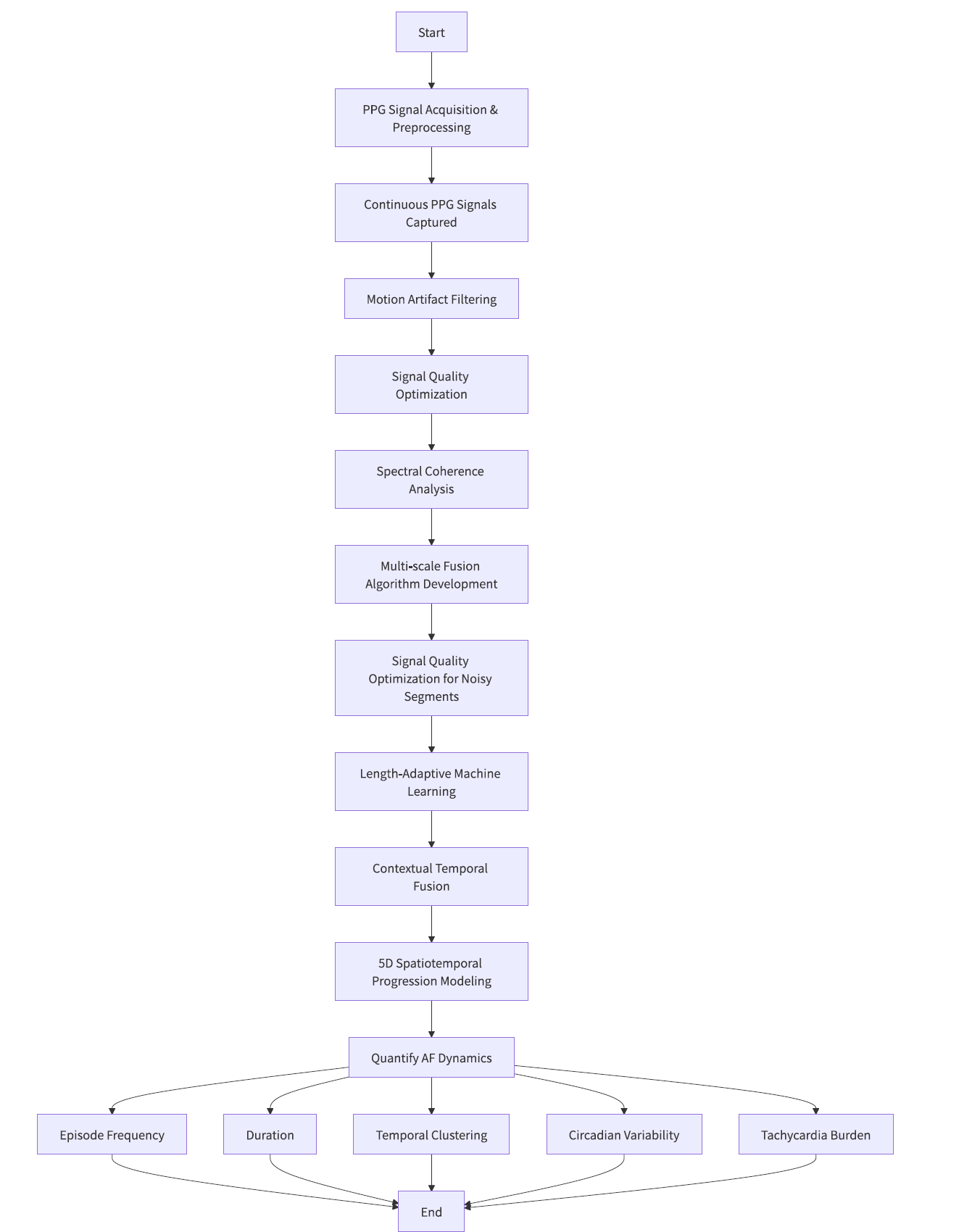


Figure S1. Schematic description of model flow.

PPG: photoplethysmography. AF: atrial fibrillation.


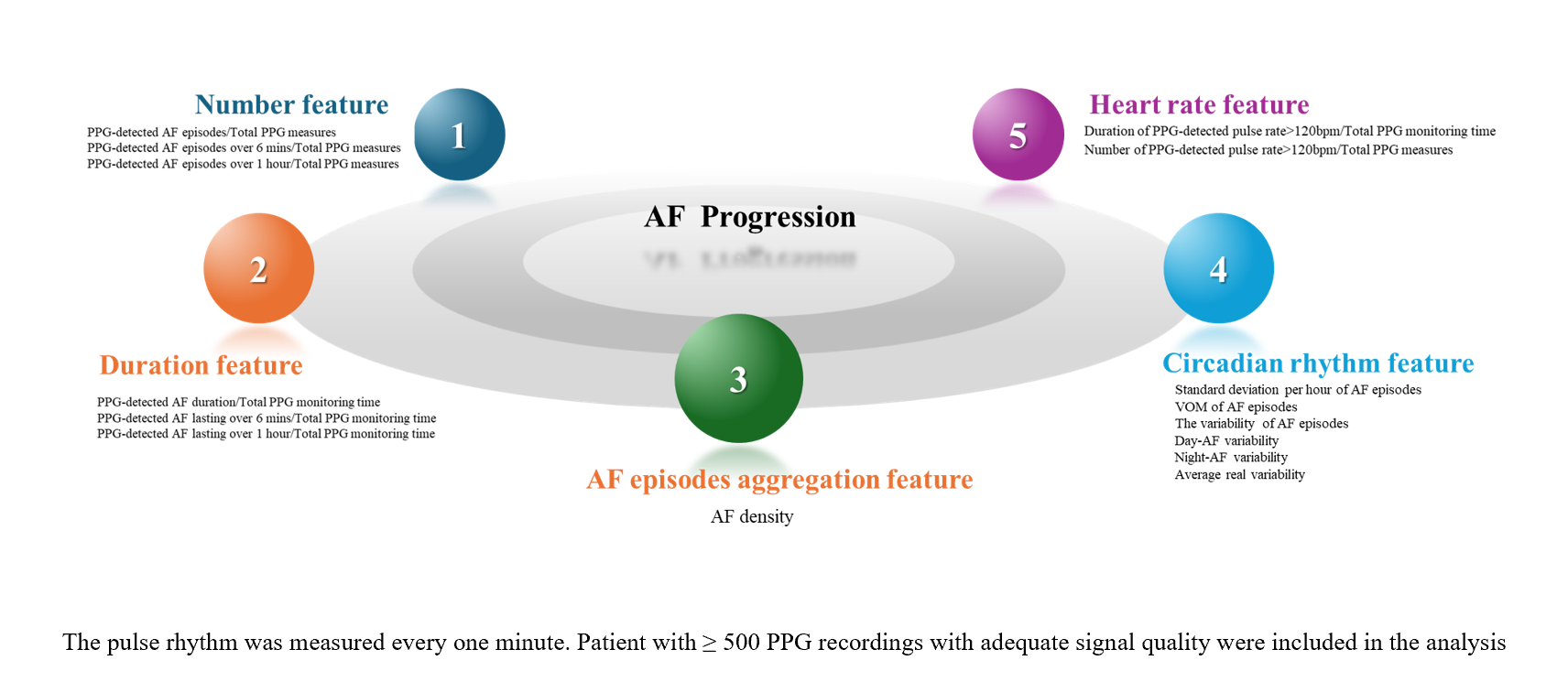


Figure S2. Multi-dimensional quantitative assessment approach of atrial fibrillation progression with photoplethysmograph-based smart watch.

PPG: photoplethysmography. AF: atrial fibrillation. VOM: the variation of the mean.


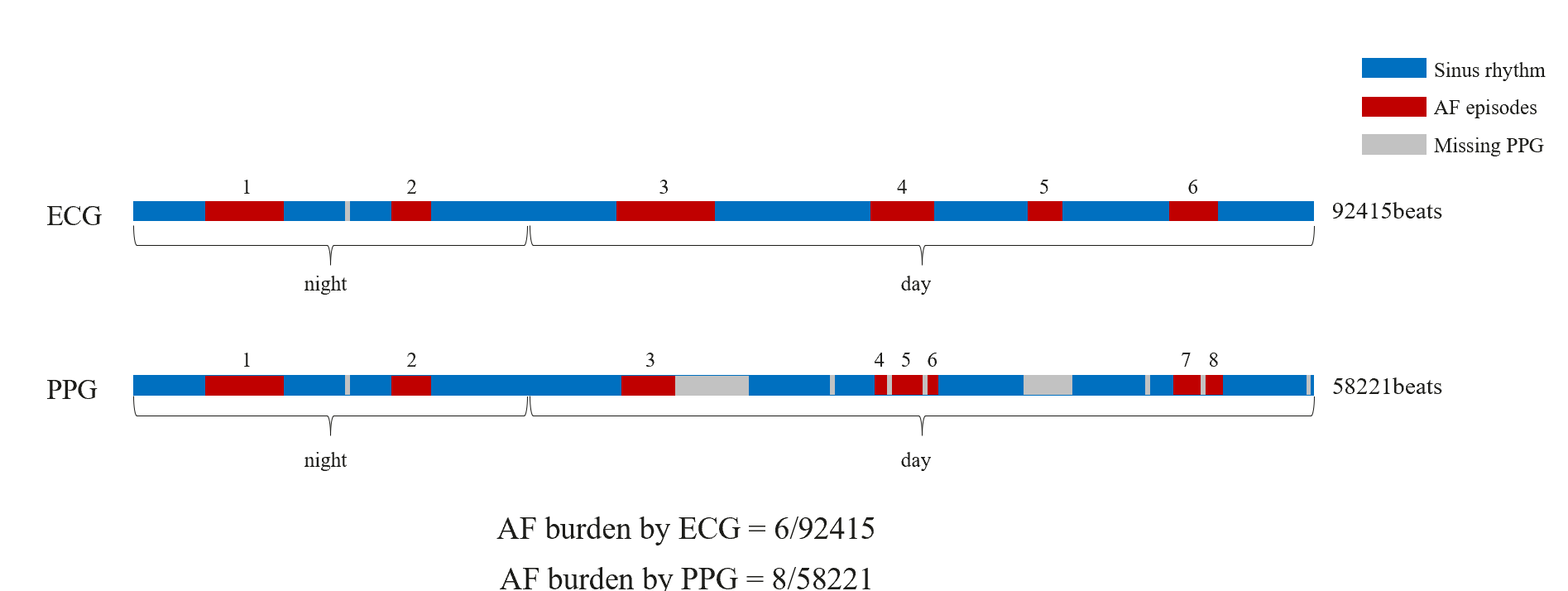


Figure S3. AF progression feature statistics schematic diagram: Episode Frequency.

PPG: photoplethysmography. AF: atrial fibrillation.


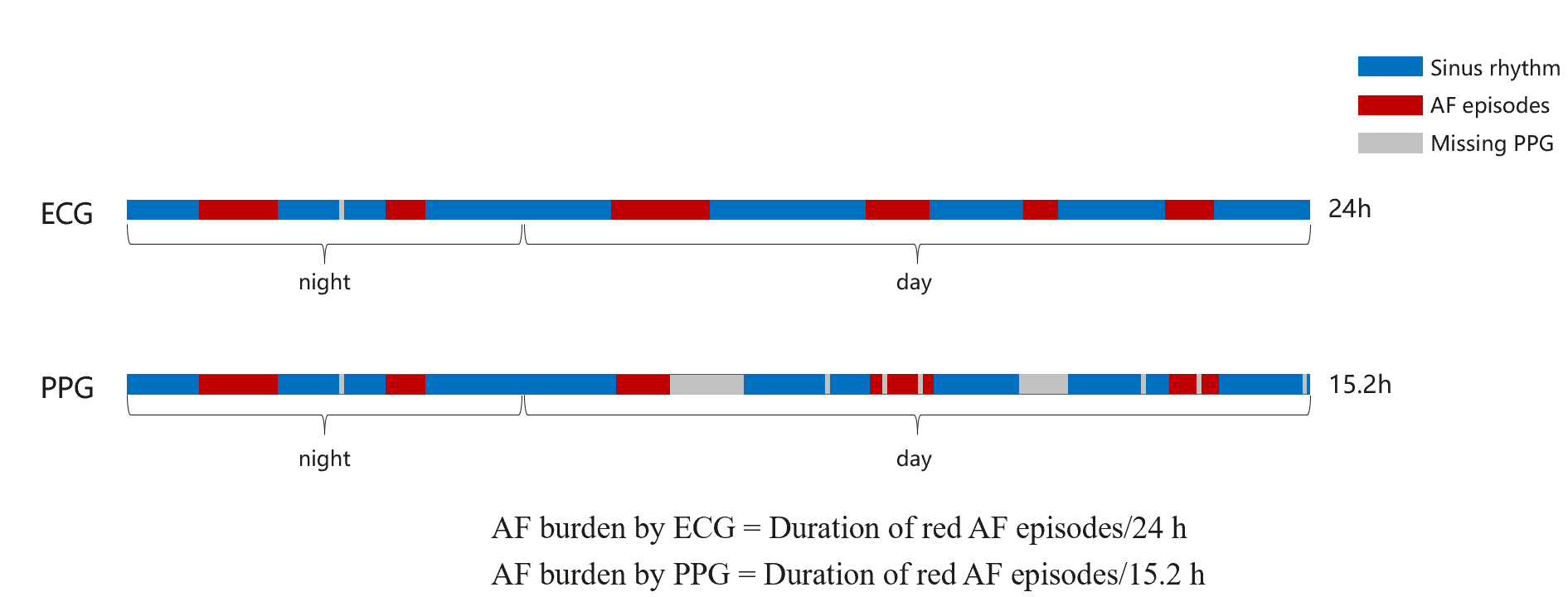


Figure S4. AF progression feature statistics schematic diagram: Duration Patterns.

PPG: photoplethysmography. AF: atrial fibrillation.


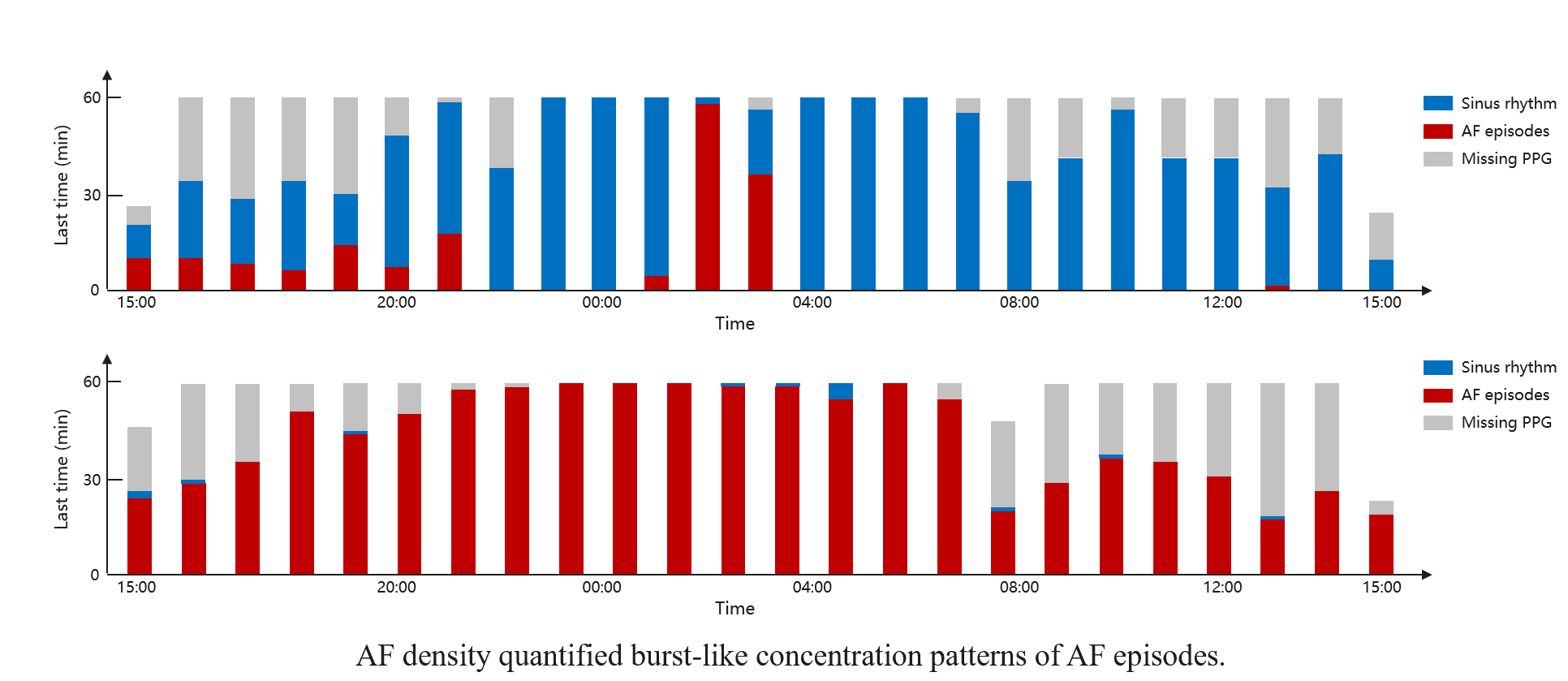


Figure S5. AF progression feature statistics schematic diagram: Temporal Clustering.

PPG: photoplethysmography. AF: atrial fibrillation.

Table S1. Patient’s characteristics at baseline.

|  | | | |
| --- | --- | --- | --- |
| **Characteristic** | |  | **Patients (n=110)** |
| Age, years, mean ± SD | |  | 62(14) |
| Male, n (%) |  |  | 72(65.45%） |
| Body mass index, kg/m2, ± SD | | | 25(4) |
| **Cardiovascular risk factors and comorbidity** | | | |
| Hyperlipidemia, n (%) | |  | 43(39.09%) |
| Diabetes, n (%) | |  | 20(18.18%) |
| Hypertension, n (%) | |  | 56(50.90%) |
| Coronary artery disease, n (%) | | | 31(28.18%) |
| Previous stroke or transient ischemic attack, n (%) | | | 11(10.00%) |
| Renal dysfunction, n (%) | | | 4(3.63%) |
| Heart failure, n (%) | |  | 3(2.73%) |
| Liver dysfunction, n (%) | | | 3(2.73%) |
| Chronic obstructive pulmonary disease, n (%) | | | 1(0.90%) |

Data are mean ± SD, or n (%). SD: standard deviation.

Table S2. Clinical visits in relation to AF treat pattern.

|  | Drug therapy (n=32) | AF ablation (n=19) | None (n=59) | p |
| --- | --- | --- | --- | --- |
| AF symptoms or heart rate issues (n=33) | 12 | 16 | 5 | <0.001 |
| Thromboembolism (n=1) | 1 | 0 | 0 | <0.001 |
| Other indications (n=11) | 5 | 2 | 4 | <0.001 |

* Compared to the patients received drug therapy or AF ablation, those without treatment had significantly fewer clinical visits for  AF symptoms, thromboembolism, etc.


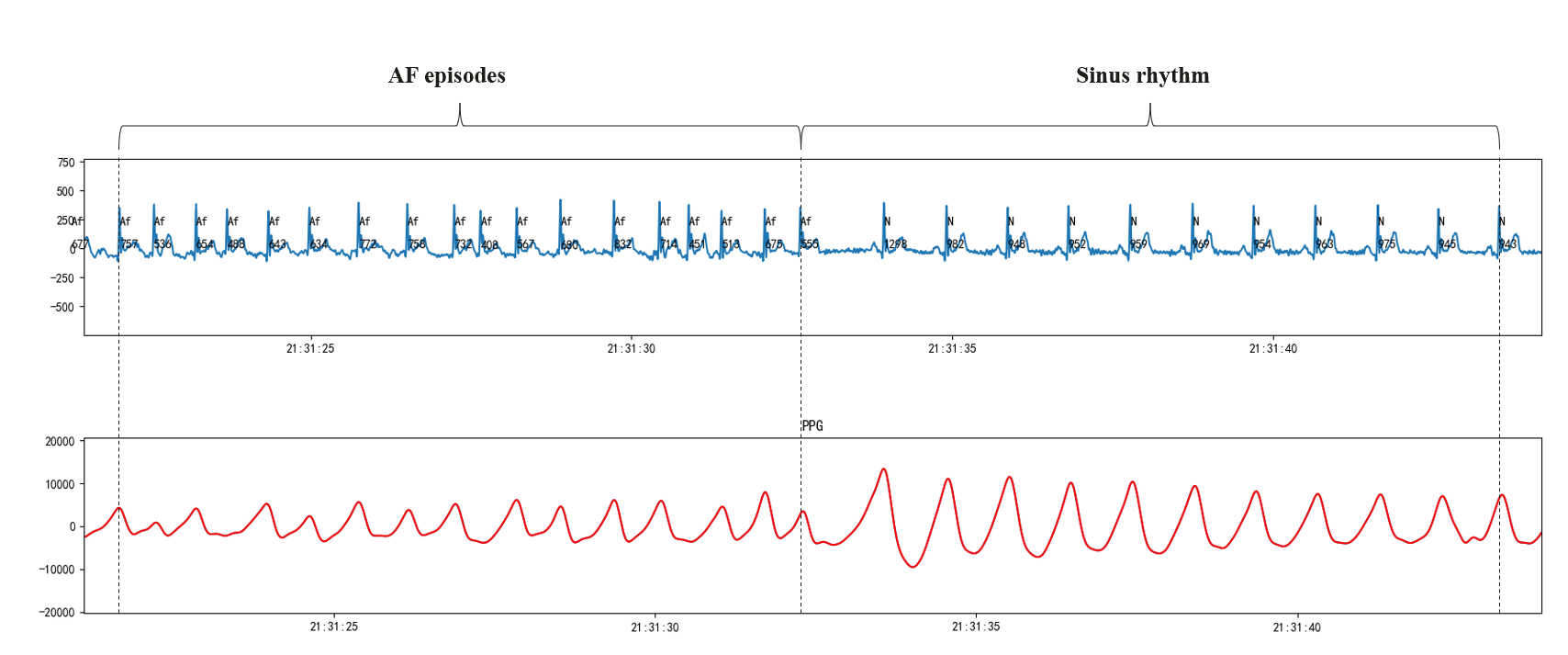


Figure S6. Comparison of ECG and PPG waveforms.

PPG: photoplethysmography. AF: atrial fibrillation.


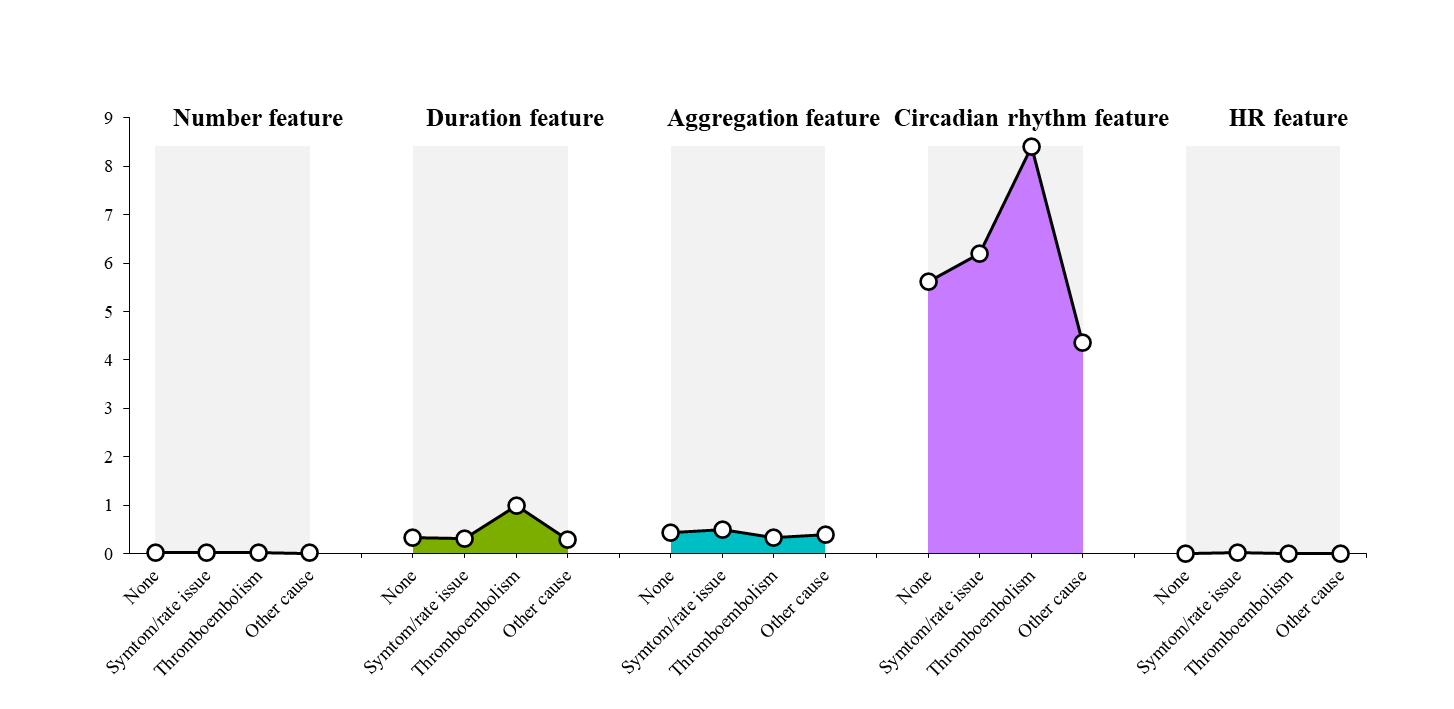


Figure S7. Clinical risk in relation to 5D spatiotemporal feature in AF progression model

Number feature：AF episodes/Total measures；Duration feature：AF duration/Total monitoring time；Aggregation feature：AF density；Circadian rhythm feature：Average real variability；HR feature：Duration of PPG-detected pulse rate > 120bpm/Total PPG monitoring time. AF: atrial fibrillation. VOM: the variation of the mean; HR: heart rate.
